# Urinary Tract Infection Caused by Cronobacter sakazakii

**DOI:** 10.7759/cureus.15780

**Published:** 2021-06-20

**Authors:** Shinichiro Hayashi, Yoshikazu Takinami, Takashi Watari

**Affiliations:** 1 Postgraduate Clinical Training Center, Shimane University Hospital, Shimane, JPN; 2 Emergency and Critical Care Medicine, Shimane University Hospital, Shimane, JPN; 3 Healthcare Quality and Safety, Harvard Medical School, Boston, USA; 4 General Medicine Center, Shimane University Hospital, Izumo, JPN

**Keywords:** cronobacter sakazakii, urinary tract infection, adult infection, gram-negative facultatively anaerobic, opportunistic infection

## Abstract

A 69-year-old man presented to the ED of our hospital with fever, loss of appetite, malaise, and pyuria and was admitted. He was diagnosed as having *Cronobacter sakazakii* urinary infection. We instituted treatment with cefmetazole, and he was discharged on hospital day 7. *C. sakazakii* is a rare infection in adults in Japan. *C. sakazakii* urinary infection usually occurs as an opportunistic infection in immunocompromised adults or older adults. This patient was not immunocompromised, so *Cronobacter *spp. should be recognized as potential pathogens in non-diabetic, non-immunocompromised adults.

## Introduction

*Cronobacter sakazakii* is a Gram-negative facultatively anaerobic rod-shaped bacterium from the Enterobacteriaceae family. The organisms previously classified as *Enterobacter sakazakii* were reassigned to the new genus *Cronobacter* in 2007 [1]. Several aspects of the pathogenicity of this organism remain unclear, although it is known to cause infections, such as colitis and meningitis in infants via ingestion of contaminated infant formula [2]. Two cases of such infection were reported in Japan in 2007 [3] and 2009 [4]. Notable infection with *C. sakazakii* in adults is rare, and no such cases have been reported in Japan. This report presents the case of an adult with a urinary tract infection (UTI) caused by *C. sakazakii*.

## Case presentation

The patient was a 69-year-old man. Six days before visiting the hospital, he experienced intermittent fever and loss of appetite. On the morning of the hospital visit, he developed severe malaise and pyuria and presented to the outpatient ED.

On arrival, he was coherent with a body temperature of 38.2°C, a blood pressure of 87/55 mmHg, oxygen saturation of 89% in room air, and bilateral costovertebral angle tenderness.

He had a history of hypertension, hyperuricemia, dyslipidemia, a burst fracture of the 12th thoracic vertebra, a vertebral body fracture of the second lumbar vertebra, ankylosing vertebral hyperostosis, angina pectoris, and surgery for a thoracic aneurysm.

Blood test results on arrival were within the reference range, except the following: platelets (107×10^3^/μL), D dimer (6.9 μg/mL), blood urea nitrogen (BUN) (55.1 mg/dL), creatinine (Crea) (2.32 mg/dL), estimated glomerular filtration rate (eGFR) (22.9 mL/min/body surface area [BSA]), C-reactive protein (CRP) (39.42 mg/dL), and procalcitonin (3.70 ng/mL). Qualitative urinalysis revealed a specific gravity of 1.021, pH of 5.5, proteinuria 2+, occult blood 3+, and urobilinogen 1+ but negative glucose, ketones, bilirubin, and nitrites. Thoracoabdominal and contrast-enhanced CT revealed a right kidney calculus and mildly increased density of perinephric adipose tissue bilaterally.

The patient was diagnosed with septic shock, and we initiated empirical antibiotic treatment with cefmetazole at 3 g/day to target *Escherichia coli*, based on the antibiotic sensitivity charts used at our hospital. Noradrenaline was administered to increase his blood pressure. His acute disseminated intravascular coagulopathy (DIC) score was 3 due to systemic inflammatory response syndrome, thrombocytopenia, and a likelihood of fibrin degradation products >10 mg/L. Therefore, the patient had not developed DIC.

On admission, the patient was markedly febrile (38.6°C), and his blood pressure was 104/51 mmHg during noradrenaline infusion (0.1 μg/kg/min). On hospital day 2, his blood pressure increased to 117/55 mmHg during noradrenaline infusion (0.02 μg/kg/min); thus, we stopped the infusion. On hospital day 5, *C. sakazakii* was detected in two sets of blood cultures and was identified as the causative pathogen. The isolate was not resistant to cephalosporins other than cefazolin, and as it was susceptible to cefmetazole, we continued the treatment. His CRP was 38.42 mg/dL on admission, which was the peak value, and subsequently decreased to 13.64 mg/dL on hospital day 5 and then normalized to 3.89 mg/dL on day 7. His blood biomarkers of renal function were abnormal on admission (BUN, 43.7 mg/dL; Crea, 1.43 mg/dL; eGFR, 38.9 mL/min/BSA) but improved as his general condition got better. By hospital day 7, the day of discharge, his BUN had decreased to 14.1 mg/dL and his Crea to 0.88 mg/dL, which indicated satisfactory renal function. The follow-up two months later showed no abnormalities.

## Discussion

This is the case that illustrates that *Cronobacter* spp. are potential UTI pathogens in non-diabetic, non-immunocompromised adults, not only for infants. *Cronobacter* spp. is well known to cause severe infections in infants. The rate of infection with *Cronobacter* is low among adults, and in adults, it usually occurs as an opportunistic infection in immunocompromised patients and older adults [2]. Infection tends to cause less severe diseases in adults than in infants [5]. According to a survey by the Foodborne Diseases Active Surveillance Network, the most common route of infection is the urinary tract in adults aged ≥40 years [6]. Infants are commonly infected after ingesting infant formula contaminated by *Cronobacter* spp.; these bacteria have been detected on organic vegetables [7]. Furthermore, according to a report by the Japanese Ministry of Health, Labour and Welfare, these organisms have been detected within the GI tract of humans and animals.

There were two possible routes of infection in the present case: oral ingestion of *C. sakazakii* from an external source, allowing the organism to enter the GI tract, and retrograde UTI. The patient in the present case was a 69-year-old man with no underlying illness who had UTI that was successfully treated with antibiotics. The strain that caused his infection appears to have been sensitive to cefmetazole, as evidenced by the improvement of the patient’s vital signs and inflammatory markers after cefmetazole administration. We had no prior experience of infection with *C. sakazakii* at our hospital, and infection with this organism is rare. Therefore, we did not perform de-escalation because of concerns about delayed resolution of the infection as a result of switching antibiotics.

## Conclusions

*Cronobacter sakazakii* is a group of Gram-negative bacteria, which has been found in a variety of dry foods, including skimmed milk powder, herbal teas, and starches, and also been found in wastewater; it is known as a pathogen to cause infections in infants via the ingestion of contaminated infant formula, but its exact pathogenesis in adults is yet to be elucidated. This case report makes it crucial for clinicians to recognize *Cronobacter* spp. as a causative pathogen of urinary infection in non-immunocompromised or non-diabetic adults.
